# Meningitic *Escherichia coli*-induced upregulation of PDGF-B and ICAM-1 aggravates blood-brain barrier disruption and neuroinflammatory response

**DOI:** 10.1186/s12974-019-1497-1

**Published:** 2019-05-15

**Authors:** Rui-Cheng Yang, Xin-Yi Qu, Si-Yu Xiao, Liang Li, Bo-Jie Xu, Ji-Yang Fu, Yu-Jin Lv, Nouman Amjad, Chen Tan, Kwang Sik Kim, Huan-Chun Chen, Xiang-Ru Wang

**Affiliations:** 1 The Cooperative Innovation Center for Sustainable Pig Production, Huazhong Agricultural University, Wuhan, 430070 Hubei China; 2 College of Veterinary Medicine, Henan University of Animal Husbandry and Economy, Zhengzhou, 450046 Henan China; 3 State Key Laboratory of Agricultural Microbiology, College of Veterinary Medicine, Huazhong Agricultural University, Wuhan, 430070 Hubei China; 4 Department of Pediatrics, Division of Pediatric Infectious Diseases, Johns Hopkins University School of Medicine, Baltimore, MD 21287 USA

**Keywords:** Blood-brain barrier, PDGF-B, ICAM-1, Permeability, Neuroinflammation

## Abstract

**Background:**

Blood-brain barrier (BBB) disruption and neuroinflammation are considered key mechanisms of pathogenic *Escherichia coli* invasion of the brain. However, the specific molecules involved in meningitic *E. coli*-induced BBB breakdown and neuroinflammatory response remain unclear. Our previous RNA-sequencing data from human brain microvascular endothelial cells (hBMECs) revealed two important host factors: platelet-derived growth factor-B (PDGF-B) and intercellular adhesion molecule-1 (ICAM-1), which were significantly upregulated in hBMECs after meningitic *E. coli* infection. Whether and how PDGF-B and ICAM-1 contribute to the development of *E. coli* meningitis are still unclear.

**Methods:**

The western blot, real-time PCR, enzyme-linked immunosorbent assay, immunohistochemistry, and immunofluorescence were applied to verify the significant induction of PDGF-B and ICAM-1 by meningitic *E. coli* in vivo and in vitro. Evan’s blue assay and electric cell-substrate impedance sensing assay were combined to identify the effects of PDGF-B on BBB permeability. The CRISPR/Cas9 technology, cell-cell adhesion assay, and electrochemiluminescence assay were used to investigate the role of ICAM-1 in neuroinflammation subversion.

**Results:**

We verified the significant induction of PDGF-B and ICAM-1 by meningitic *E. coli* in mouse as well as monolayer hBMECs models. Functionally, we showed that the increase of PDGF-B may directly enhance the BBB permeability by decreasing the expression of tight junction proteins, and the upregulation of ICAM-1 contributed to neutrophils or monocytes recruitment as well as neuroinflammation subversion in response to meningitic *E. coli* infection.

**Conclusions:**

Our findings demonstrated the roles of PDGF-B and ICAM-1 in mediating bacterial-induced BBB damage as well as neuroinflammation, providing new concepts and potential targets for future prevention and treatment of bacterial meningitis.

## Background

Although substantial progress has been made in prevention and therapy for infectious diseases in recent years, bacterial meningitis remains an important issue in terms of infection-caused death worldwide [1]. *Haemophilus influenzae* type B, *Neisseria meningitidis*, *Streptococcus pneumoniae*, group B *streptococcus*, *Escherichia coli*, and *Staphylococcus aureus* are the most common causes of meningitis in newborns and adults [2–5]. Among these, pathogenic *E. coli*, an important zoonotic bacterial pathogen, has been widely reported to invade the host central nervous system (CNS), causing severe intracranial infection with high mortality, as well as neurodegenerative disorders and sequelae [6]. However, the mechanisms by which meningitic *E. coli* enters the host brain and causes neuroinflammatory responses are poorly understood. Identification of the specific host molecules involved in these essential steps is urgently needed to clarify these mechanisms.

The blood-brain barrier (BBB) consists of brain microvascular endothelial cells (BMECs), pericytes, and astrocytes and separates the brain parenchyma from blood circulation. The BBB ensures the brain’s energy supply and stabilizes the microenvironment by discharging CNS metabolites and neurotoxic agents into circulation, while preventing circulating neurotoxic agents, inflammatory factors, or immune cells entering the CNS [7]. BMECs are the most direct and functional structural component of the BBB and are closely linked by the expression of tight junction (TJ) proteins, including Claudins, Occludin, and cytoplasmic zonala-occludin family members (such as ZO-1, ZO-2, and ZO-3) [8]. Decrease or distribution changes of these TJ proteins could lead to increased BBB permeability, which is an important indicator of BBB dysfunction [9].

Platelet-derived growth factor (PDGF) is a family of growth factors comprising five dimeric ligands (PDGF-AA, PDGF-AB, PDGF-BB, PDGF-CC, and PDGF-DD) assembled from four gene products (PDGF-A, PDGF-B, PDGF-C, PDGF-D) [10]. Members of this family are disulfide-bonded polypeptides with multifunctional roles, including inflammation, brain development, and vascular permeability [11, 12]. Notably, increasing studies support the major role of PDGF-BB in a variety of disease models [13]. In tumor tissues, the overexpression of PDGF-BB has been reported to promote tumor angiogenesis [14]. In chronic hepatitis C virus infection, the induction of PDGF-BB mediated hepatic stellate cell proliferation, leading to liver fibrosis and cirrhosis [15]. Another recent study reported that HIV infection can exploit host PDGF-BB to break the integrity of the BBB by decreasing TJ protein expression [16]. Moreover, a stroke model study reported that the abnormal expression of PDGF-BB can enhance the permeability of BBB [17]. Despite these, no previous studies have investigated the role of PDGF-BB in meningitic *E. coli* penetration of the BBB. Our previous RNA-sequencing data revealed the significant upregulation of PDGF-BB in human BMECs (hBMECs) upon meningitic *E. coli* challenge [18], implying a specific role of this molecule in the infection process. However, the specific effects of PDGF-BB on meningitic *E. coli* penetration of the BBB remain unclear.

Intercellular adhesion molecule-1 (ICAM-1) is a member of the immunoglobulin superfamily cell adhesion molecule [19], which is originally constitutively expressed in endothelial cells at low levels but can be highly induced in response to certain inflammatory and infectious conditions, leading to secretion of chemokines and cytokines such as monocyte chemoattractant protein-1 (MCP-1), interleukin-1 beta (IL-1β), and TNFα [20]. ICAM-1 is now widely considered to be an important marker for endothelium activation, and its canonical function is widely believed to promote migration of leukocytes or monocytes to the infection site [21]. ICAM-1 is reported to be highly induced in response to a variety of pathogenic microbes, such as *Plasmodium falciparum* [22] and *E. coli* [23], and has been identified as a receptor for virus infection, such as *rhinoviruses* [24]. In a previous study of transcriptomics data, we found that ICAM-1 was significantly increased in hBMECs upon meningitic *E. coli* infection [18], indicating activation of BMECs. However, the specific function of ICAM-1 in this situation remains to be characterized.

In the current study, to further explore the pathogenic mechanisms of meningitic *E. coli*-induced CNS infection, we characterized two key molecules, PDGF-BB and ICAM-1, in hBMECs in response to meningitic *E. coli*, and investigated their potential roles in mediating BBB permeability alteration as well as neuroinflammatory response. Our in vivo and in vitro results largely supported the contribution of PDGF-BB to BBB permeability via decreasing TJ proteins and the ICAM-1-initiated immune response by recruiting leukocyte or monocyte migration and traversing the BBB. PDGF-BB, as well as ICAM-1, are therefore likely to represent important candidate targets, which may provide novel insights for future prevention and non-antibiotic therapy for pathogenic *E. coli* meningitis.

## Methods

### Bacterial strains, cell culture, and reagents

Meningitic *E. coli* strain PCN033 used herein was originally isolated from pig cerebrospinal fluid from a diseased farm in China, 2006 [25], and was kept in our laboratory. Bacterial cells were routinely grown in Luria-Bertani medium at 37 °C.

The hBMEC cell line was routinely cultured in RPMI1640 medium supplemented with 10% fetal bovine serum (FBS), 10% Nu-Serum, 2-mM L-glutamine, 1-mM sodium pyruvate, nonessential amino acids, vitamins, and penicillin and streptomycin (100 U/mL) in a 37 °C incubator under 5% CO_2_ until monolayer confluence [26]. Confluent cells were washed with Hanks’ balanced salt solution and starved in serum-free medium (1:1 mixture of Ham’s F-12 and M-199) for 16–18 h before the experiment. The human monocyte cell line THP-1 was commercially purchased from ATCC (TIB-202) and cultured in RPMI1640 medium supplemented with 2-mM L-glutamine and 10% FBS [27].

### Reagents and antibodies

Evan’s blue dye was purchased from Santa Cruz Biotechnology (Santa Cruz, CA, USA). Recombinant human PDGF-BB was obtained from Novoprotein (Summit, NJ, USA) and recombinant mouse PDGF-BB was obtained from Novus Biologicals (Littleton, CO, USA). Mouse PDGF-BB ELISA Kit was purchased from 4A Biotech, Co., Ltd (Beijing, China). The ICAM-1 neutralizing antibody was obtained from Abcam (Cambridge, MA, USA). For western blot analysis, PDGF-BB antibody (rabbit) was obtained from GeneTex (Irvine, CA, USA) and PDGF-B antibody (rabbit) was obtained from Abcam. ICAM-1 (rabbit) and ZO-1 (rabbit) antibodies were obtained from Cell Signaling Technology (Danvers, MA, USA). Claudin-5 (rabbit) and Occludin (rabbit) antibodies were obtained from Arigo Biolaboratories (Hsinchu, Taiwan). The β-actin (mouse) antibody was obtained from Proteintech (Chicago, IL, USA). The super electrochemiluminescence (ECL) kit was obtained from US Everbright Inc. (Suzhou, China). For immunohistochemistry and immunofluorescence, PDGF-BB antibody (rabbit) was obtained from GeneTex (Irvine, CA, USA) and Claudin-5 antibody (rabbit) was obtained from Abcam (Cambridge, MA, USA). ICAM-1 (rabbit), ZO-1 (rabbit), Occludin (rabbit), and CD34 (mouse) antibody were obtained from Proteintech (Chicago, IL, USA). FITC-labeled goat anti-mouse and Cy3-labeled goat anti-rabbit antibodies and DAPI were obtained from Beyotime Institute of Biotechnology (China). A human pYSY-spCas9-sgRNA-Puro vector containing the Cas9 product and the guide RNA sequence was obtained from YSY Biotech (Nanjing, China).

### Meningitic *E. coli* infection of hBMECs

*E. coli* strain PCN033 infection of hBMECs was performed following previously described methods [28]. Briefly, overnight cultures of *E. coli* were resuspended and diluted in serum-free medium and then added to the confluent hBMECs monolayer grown in 100-mm dishes at a multiplicity of infection of 10 (approximately 10^8^ colony-forming units (CFUs) per dish) to allow invasion at 37 °C. Finally, cells were washed three times with pre-chilled PBS and subjected to RNA extraction using TRIzol reagent or using cell lysis buffer for western blot analysis. In some assays, the supernatant of cell culture was collected and concentrated for immunoblotting of PDGF-BB.

### Reverse transcription and real-time polymerase chain reaction (PCR)

TRIzol reagent (Aidlab Biotech, Beijing, China) was used to isolate total RNA from the infected hBMECs. Aliquots (500 ng) of the total RNA in each sample were subjected to cDNA synthesis using the HiScript II Q RT SuperMix for qPCR gDNA wiper (Vazyme, Nanjing, China). Primers for the quantitation real-time PCR were as follows: PDGFB, 5′-GCTCTTCCTGTCTCTCTG-3′ (forward) and 5′-GGTCACTCAGCATCTCATA-3′ (reverse); ICAM-1, 5′-GTAGCAGCCGCAGTCATAA-3′ (forward) and 5′-GCCTGTT GTAGTCTGTATTTCTTG-3′ (reverse); ZO-1, 5′-TGTGGAAGAGGATGAAGATG AAGA-3′ (forward) and 5′-GGTGGAAGGATGCTGTTGTC-3′ (reverse); occludin, 5′-TTAACTTCGCCTGTGGAT-3′ (forward) and 5′-TGTGTAGTCTGTCTCATAGTG-3′ (reverse); Claudin-5, 5′-CGCCTTCCTGGACCACAACAT-3′ (forward) and 5′-CCAG CACCGAGTCGTACACTT-3′ (reverse); and GAPDH, 5′-TGCCTCCTGCACCACCAAC T-3′ (forward) and 5′-CGCCTGCTTCACCACCTTC-3′ (reverse). Real-time PCR was performed with the real-time PCR thermal cycler qTOWER^3^ (Analytik Jena, Germany) using BioEasy SYBR Green master mix (Bioer Technology, Hangzhou, China) according to the manufacturers’ recommendations. The transcriptional levels of the target mRNA were normalized to GAPDH. Data were presented as mean ± SD from three independent assays.

### Western blot analysis

Mouse brains and hBMECs were homogenized or lysed in RIPA buffer with protease inhibitor cocktail and centrifuged at 12,000 rpm for 10 min at 4 °C to remove the insoluble cell debris. For the secretory PDGF-BB analysis, cell culture supernatant (90 mL for each sample) was collected and subjected to a concentration process by centrifugation at 4,000 rpm for 10 min at 4 °C as well as 0.22-μm filter to remove the possible cell and bacterial debris. The supernatant was then added with 10 mL of 15% trichloroacetic acid and incubated at 4 °C overnight. The mixture was then centrifuged at 13,000 rpm for 15 min at 4 C to remove the supernatant, and the precipitate was washed twice with acetone. The protein concentrations from brain lysates, cell lysates, or the concentrated supernatant were measured with a BCA protein assay kit (NCM Biotech, China) and applied to western blot analyses. The densitometry analysis was performed using ImageLab software version 5.2.1 (Bio-Rad, Hercules, CA, USA).

### Secretory cytokines determination by enzyme-linked immunosorbent assay (ELISA)

Mice were challenged with bacteria, and serum and brains were collected as mentioned above. Secretory PDGF-BB from serum and brain tissue was quantified using a PDGF-BB ELISA Kit, as mentioned in the “Methods” section, following the manufacturers’ instructions.

### Electrochemiluminescence assay

For ICAM-1 neutralizing assay in vivo, the ICAM-1 neutralizing antibody, purchased from Abcam (Cambridge, MA, USA), was injected intravenously at the dosage of 50 μg per mouse 2 h prior to bacterial challenge. The mice in the control group were injected with the equivalent bovine serum albumin (BSA). Mice were then intravenously challenged with 100 μL of bacterial suspension containing 1 × 10^7^ CFUs. After 6 h of infection, mice were subjected to euthanasia and brain lysates and serum were harvested for IL-1β and TNF-α quantitation using ECL assay, following the manufacturer’s instructions (Meso Scale Discovery, Meso Scale Diagnostics, Rockville, MD, USA).

### Immunohistochemistry and immunofluorescence analysis

For immunohistochemistry IHC, paraffin sections were deparaffinized and rehydrated in xylene and ethanol. Endogenous peroxidase was quenched by incubation in 3% hydrogen peroxide, and antigen retrieval was performed in a 10-mM citrate buffer. Sections were then blocked with 5% BSA in PBS for 1 h at room temperature followed by incubation with primary antibody at 4 °C overnight. After washing with PBS, the secondary antibody was applied and diaminobenzidine (DAB) was added for color development. For immunofluorescence (IF), sections were incubated with the primary antibody, followed by incubation with secondary antibody conjugated with either Cy3 or FITC. The same sections were then incubated with CD34 primary antibody, followed by incubation with the appropriate secondary antibody prior to the final nucleus staining with DAPI. Sections were photographed and analyzed using BX41 Microscopy (Olympus, Tokyo, Japan).

### Animal infection assay

The 25-day-old SPF KM mice (female) were obtained from the experimental animal center at China Three Gorges University (Hubei Province, China) for animal infection assays. Mice were challenged with *E. coli* strain PCN033 through the tail vein at 1 × 10^7^ CFUs suspended and diluted in phosphate-buffered saline (PBS; pH 7.4). The mild symptoms of mice were defined as dull and anorexic (appearing approximately 2–3 h post infection); the moderate symptoms of mice were defined as depressed, dull, and somnolent (approximately 5–6 h post infection); the severe symptoms of mice were defined as trembling and paddling (around 7–8 h post infection); and moribund mice behaved as overexcited and opisthotonus (approximately 9–12 h post infection). Mice with different symptoms were anesthetized for collection of the peripheral blood, and then perfused with heparin sodium solution (10 U/mL) in PBS as previously described [29]. The brains were finally collected and processed for further assays.

For PDGF-BB, three samples (including mouse brain and serum) from each mice group with similar symptoms were randomly selected for ELISA determination, and the brains from moribund mice were moreover subjected to the IF and IHC assays. For ICAM-1, three brains in each group with similar symptoms were randomly pooled for western blot analysis, and the brains from mice with mild symptoms were moreover subjected to IF and IHC assays. For ECL assay, the brains and serum were collected after 6 h of infection. In the survival assay, mice were pretreated via tail vein injection with or without 50 μg ICAM-1 neutralizing antibody (Abcam, Cambridge, MA, USA) for 2 h prior to the challenge, and the fatality of the mice in each group was recorded.

### In vivo blood-brain barrier permeability assay

BBB permeability was evaluated using Evan’s blue dye (961 Da), which binds to intravascular serum albumin in vivo to become a protein tracer with high molecular weight [30]. Briefly, recombinant mouse PDGF-BB (10, 100, and 500 ng/mouse) was pre-injected intravenously for up to 24 h, and 500-μL Evan’s blue (5 mg/mL) was injected via the tail vein to allow circulation for 10 min before the mice were sacrificed and perfused. Brains were then taken and photographed for extravascular staining of the dye.

### Electric cell-substrate impedance sensing

To explore the function of PDGF-BB in permeability of the BBB, hBMECs were seeded on collagen-coated, gold-plated electrodes in 96-well chamber slides (96W1E+) linked to electric cell-substrate impedance sensing (ECIS) Zθ equipment (Applied BioPhysics, Troy, NY) and monitored continuously to reflect any alterations of the barrier function. After stable maximal trans-endothelial electric resistance (TEER) was reached, the recombinant human PDGF-BB protein was added into the cells at a specified dosage (2, 5, 10, and 20 ng/mL). TEER changes of the monolayer cells were automatically recorded with the ECIS system.

### Deletion of ICAM-1 in hBMECs via CRISPR/Cas9 technology

An all-in-one pYSY-spCas9-sgRNA-Puro vector was obtained from YSY Biotech (Nanjing, China). Human ICAM-1 sgRNA1 (5′-CCTGCCTGGGAACAACCGGA-3′) and sgRNA2 (5′-TCAAAAGTCATCCTGCCCCG-3′) were synthesized then cloned into the all-in-one vector to generate the pYSY-spCas9-ICAM-1-sgRNA-Puro plasmid. hBMECs were seeded into six-well plates at a density of 2 × 10^5^ cells per well for 24 h followed by the transient transfection with a 2-μg pYSY-spCas9-ICAM-1-sgRNA-Puro plasmid using Lipofectamine 3000 (Life Technologies). The cells were incubated at 37 °C with 5% CO_2_ for 24 h, and fresh medium containing 200 ng/mL puromycin was added and incubated for another 48 h. Cells were then collected and each single-cell clone was transferred into 96-well plates via a limiting dilution method. Genomic DNA was extracted when cells were confluent using QuickExtract DNA Extraction Solution (YSY Biotech, Nanjing, China). PCR was performed to amplify the target region with the following primers: 5′-TCCACATCGAAGGCAAAGTA-3′ (forward) and 5′-CTACGAGCAAGTGGCAAAGA-3′ (reverse). Cells with ICAM-1 sequence deletion were validated through sequencing as well as ICAM-1 expression via western blot analysis.

### Cell-cell adhesion assay

Adherence of THP-1 monocytes to endothelial cells was evaluated using fluorescence-labeled monocytes according to previously described methods [31]. In brief, monocytes were labeled with 5-μM Vybrant DiD (Sigma-Aldrich, USA) for 20 min at 37 °C in phenol red-free RPMI1640 medium containing 5% FBS. Fluorescence of labeled cells was measured (total signal) with a fluorescence microplate reader (TECAN, Mannedorf, Switzerland). After washing twice, the cells (1.5 × 10^6^/mL, 200 μL/well) were suspended in adhesion medium (RPMI1640 containing 2% FBS and 20-mM HEPES) and added to confluent monolayers of wide-type hBMECs or ICAM-1 knocking-out cells in 96-well plates which were pre-challenged for 2 h with meningitic *E. coli*. After incubation for 1 h at 37 °C, the non-adherent free monocytes were removed by washing four times, and fluorescence signals of adherent cells were measured as described previously [31]. Monocyte adherence was presented as the percentage adherence and calculated as (adherent signal/total signal) × 100. Results were shown based on data from three independent replicates.

### Statistical analysis

Data were expressed as mean ± standard deviation (mean ± SD), and the significance of differences between groups was evaluated using one-way analysis of variance or log-rank (Mantel-Cox) test. A level of *p* < 0.05 (*) was considered significant, and *p* < 0.01 (**) or *p* < 0.001 (***) was considered extremely significant. Graphs were plotted and analyzed using GraphPad Prism Ver. 6.0 (GraphPad Software, La Jolla, CA, USA).

## Results

### Meningitic *E. coli* significantly stimulates the production and secretion of PDGF-BB in vivo and in vitro

**Fig. 1:**
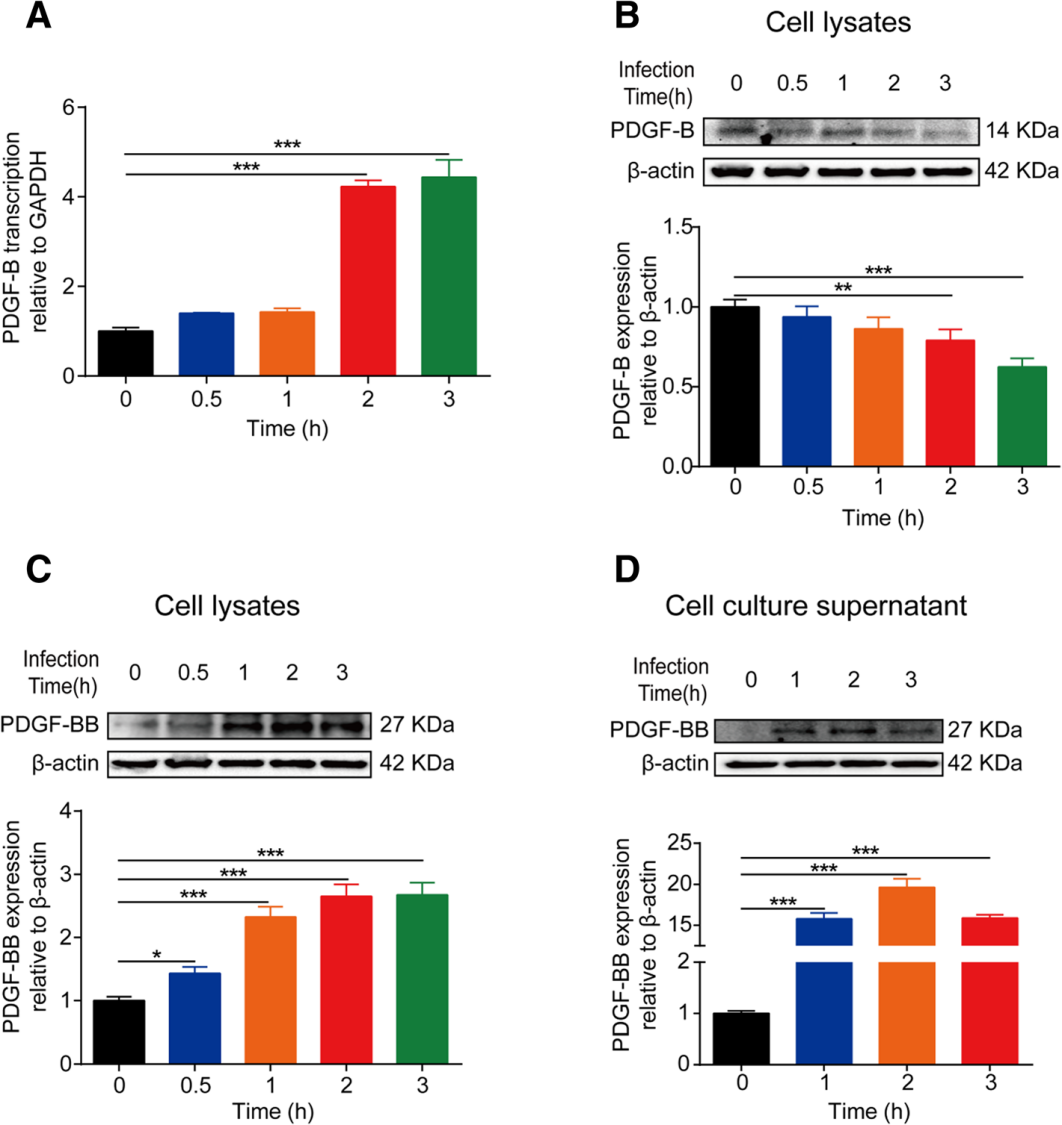
Meningitic *E. coli* infection increases production as well as secretion of PDGF-BB in hBMECs. **a** Meningitic *E. coli* PCN033 induced a time-dependent increase of PDGF-B transcription in hBMECs. GAPDH was used as the reference control. **b** Western blot analysis revealed a significant decrease of PDGF-B protein in the lysates of hBMECs in response to infection. β-actin was used as a loading control, and densitometry was performed to analyze differences among the samples. **c**, **d** Western blot analysis of the PDGF-BB dimer in either lysates or the concentrated culture supernatant of hBMECs after infection. β-actin was used as the loading control, and densitometry was performed to analyze differences

**Fig. 2:**
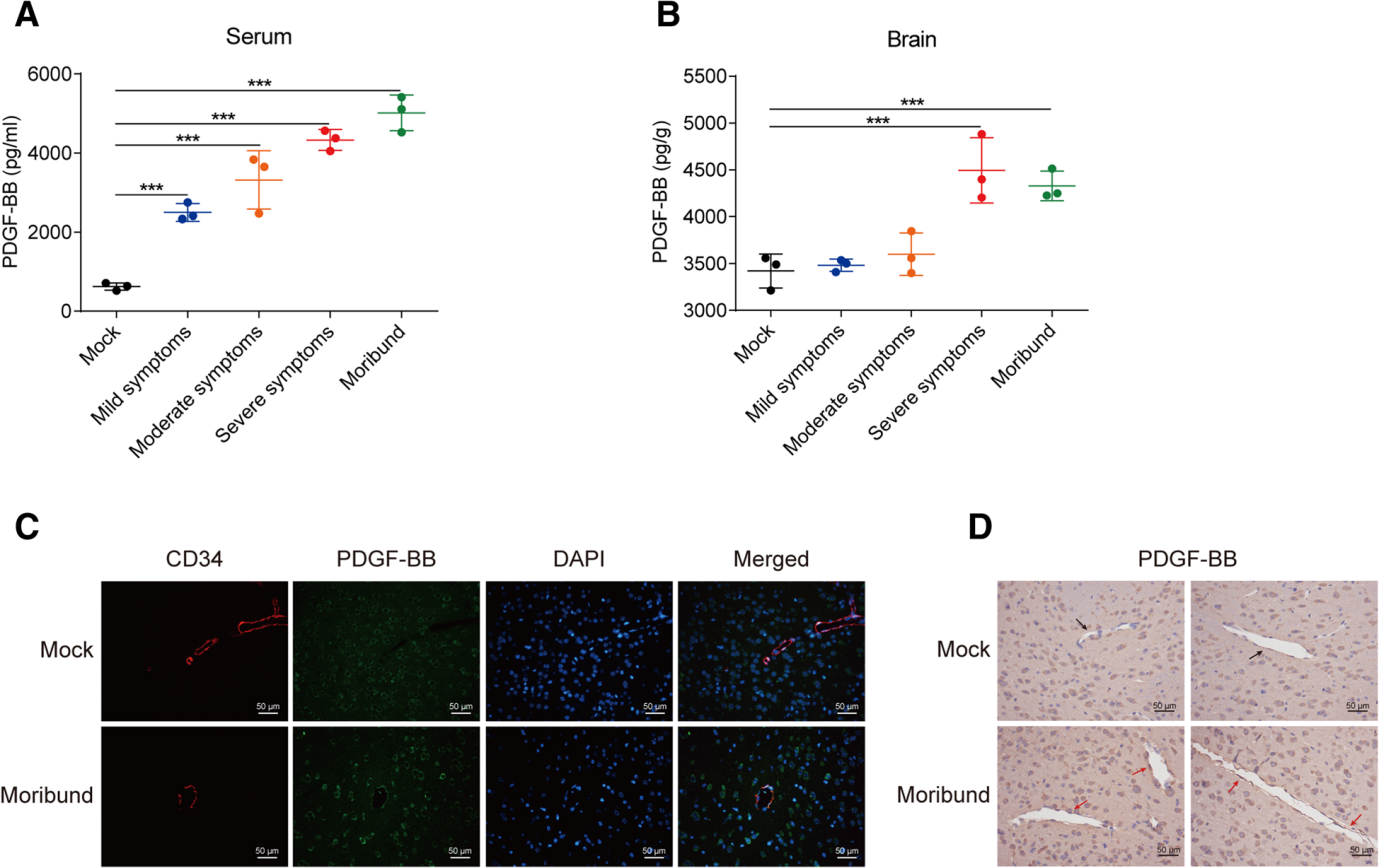
PDGF-BB was upregulated in meningitic *E. coli*-challenged mice. **a**, **b** ELISA analysis of PDGF-BB in serum and brain lysates from challenged mice exhibiting different symptoms (*n* = 3). Data are presented as mean ± SD from three individual mice in each group. **c** The brains of moribund and control mice were taken and analyzed for the expression of PDGF-BB via IF. PCN033-induced perivascular PDGF-BB was labeled in green. CD34 was specifically applied for labeling the blood vessels in red. The cell nucleus was stained in blue with DAPI. Scale bar indicates 50 μM. **d** The brains of moribund and control mice were subjected to PDGF-BB expression by IHC. PDGF-BB around the vessels was indicated by red arrows (infection group) or black arrows (mock group). Scale bar indicates 50 μM

To verify our earlier RNA-Seq data that PDGF-B was significantly induced in response to meningitic *E. coli*, both in vivo and in vitro BBB models were applied to examine the expression changes of PDGF-B. In the monolayer hBMEC model, using real-time PCR, the transcription of PDGF-B was significantly and time-dependently increased with the bacterial challenge (Fig. 1a). But at the protein level, the results revealed that the expression of PDGF-B decreased along with infection (Fig. 1b). Since PDGF-B is initially transcribed in its monomeric form, and turns into a homodimer complex (PDGF-BB) to perform its function, we next analyzed the expression of PDGF-BB during this infection course. As expected in Fig. 1c, the PDGF-BB protein was shown to be significantly increased in cell lysates in a time-dependent manner, and the secreted PDGF-BB in culture supernatant was also obviously increased (Fig. 1d). Likewise, in mice infected by meningitic *E. coli*, the circulatory PDGF-BB in the serum exhibited a prominent increase accompanying with the infection course (Fig. 2a). Meanwhile, secreted PDGF-BB was also observed in the brain, exhibiting a significant increase, with a significantly higher level of PDGF-BB in the brains of mice with severe symptoms or those that were moribund compared with mice with mild symptoms, moderate symptoms, or control mice (Fig. 2b). In addition, the moribund mice brains were collected and the IF and IHC were performed to further analyze the PDGF-BB expression in situ. As shown in Fig. 2c and d, compared with control mouse brain, PDGF-BB was increased and more distributed around the blood vessels in the brains of moribund mice. Taken together, these in vitro and in vivo results indicate that meningitic *E. coli* infection can induce significant production and secretion of PDGF-BB.

### PDGF-BB enhances BBB permeability by decreasing the expression of TJ proteins

**Fig. 3:**
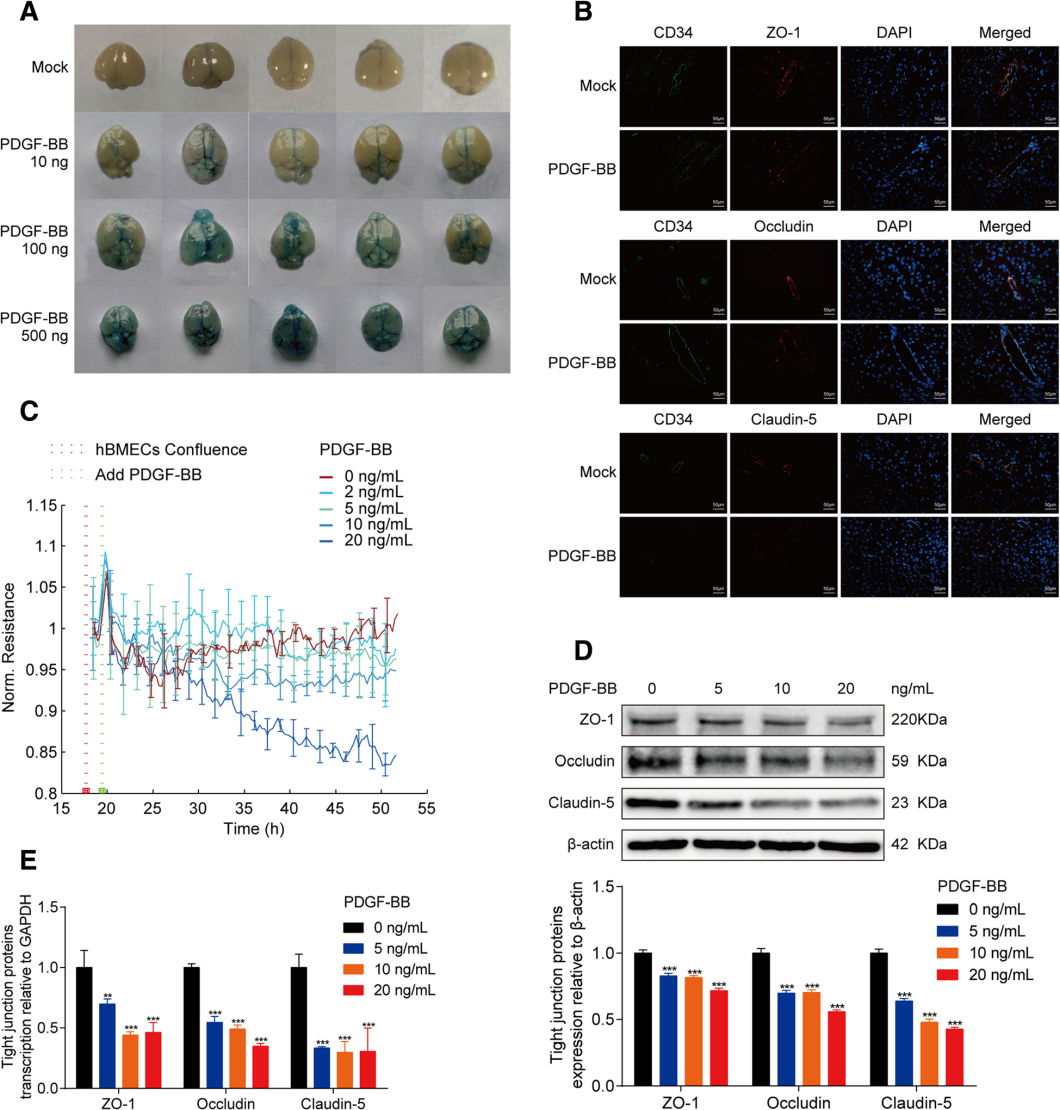
PDGF-BB contributes to BBB disruption by downregulating TJ proteins. **a** Effects of the recombinant mouse PDGF-BB (10, 100, and 500 ng/mouse) on permeability of the mouse brain evaluated by Evan’s blue approach. **b** Mice were treated with or without PDGF-BB, and brains from both groups of mice were analyzed for the integrity of vascular endothelium by IF. The TJ proteins ZO-1, Occludin, and Claudin-5 were selected as the markers reflecting the integrity of the vascular endothelium. CD34 was specifically applied for labeling the microvessels. Scale bar indicates 50 μm. **c** Recombinant PDGF-BB treatment causes a significant and dose-dependent decrease of monolayer TEER based on ECIS system. Data were presented as mean ± SD from three independent assays. **d** Western blot analysis of ZO-1, Occludin, and Claudin-5 in hBMECs in response to multiple dosage of PDGF-BB (0, 5, 10, and 20 ng/mL). β-actin was used as the loading control, and densitometry was performed to analyze the differences. **e** Real-time PCR analysis of ZO-1, Occludin, and Claudin-5 transcription in hBMECs treated by multiple dosages of PDGF-BB. GAPDH was used as the internal reference. Data were presented as mean ± SD from three independent assays

Since a high level of PDGF-BB was detected in mouse serum and brain tissue after infection, we next investigated the possible influence of PDGF-BB on BBB function using intravenous (tail vein) injection of recombinant mouse PDGF-BB (10, 100, and 500 ng/mouse). As shown, injection of recombinant PDGF-BB resulted in a dose-dependent increase of the BBB permeability. The injected Evan’s blue dye largely leaked out of the blood vessels in both 100-ng and 500-ng PDGF-BB-treated mouse brains compared with the mock control (Fig. 3a), reflecting BBB damage in the brain. Meanwhile, the immunofluorescence (IF) was performed to examine the distribution of TJ proteins (ZO-1, Occludin, and Claudin-5) in mice brain in response to PDGF-BB treatment for 24 h. As shown in Fig. 3b, these TJ proteins were found to be well-organized and distributed around the blood vessels in the control mice brain. In contrast, the vascular endothelial layer became inconsecutively distributed, irregular or gapped in PDGF-BB-treated mice, indicating a breakdown of the TJ proteins between adjacent endothelial cells. We additionally evaluated the possible effects of PDGF-BB on TEER of the hBMEC monolayer in vitro through ECIS system. The results indicated that PDGF-BB treatment significantly decreased the TEER values of the hBMEC monolayer dose-dependently (Fig. 3c), in accordance with our in vivo observations, suggesting that PDGF-BB could be an important contributor to the BBB damage. Moreover, by using different dosages of recombinant human PDGF-BB to stimulate hBMECs for 24 h, we found that the expression of TJ proteins including ZO-1, Occludin, and Claudin-5 were significantly decreased (Fig. 3d), and their mRNA levels were also dose-dependently downregulated in response to PDGF-BB (Fig. 3e). Taken together, these observations indicate that meningitic *E. coli*-induced production of PDGF-BB is an important contributor to BBB breakdown via downregulating the TJ proteins.

### Significant induction of ICAM-1 by meningitic *E. coli* infection in vivo and in vitro

**Fig. 4:**
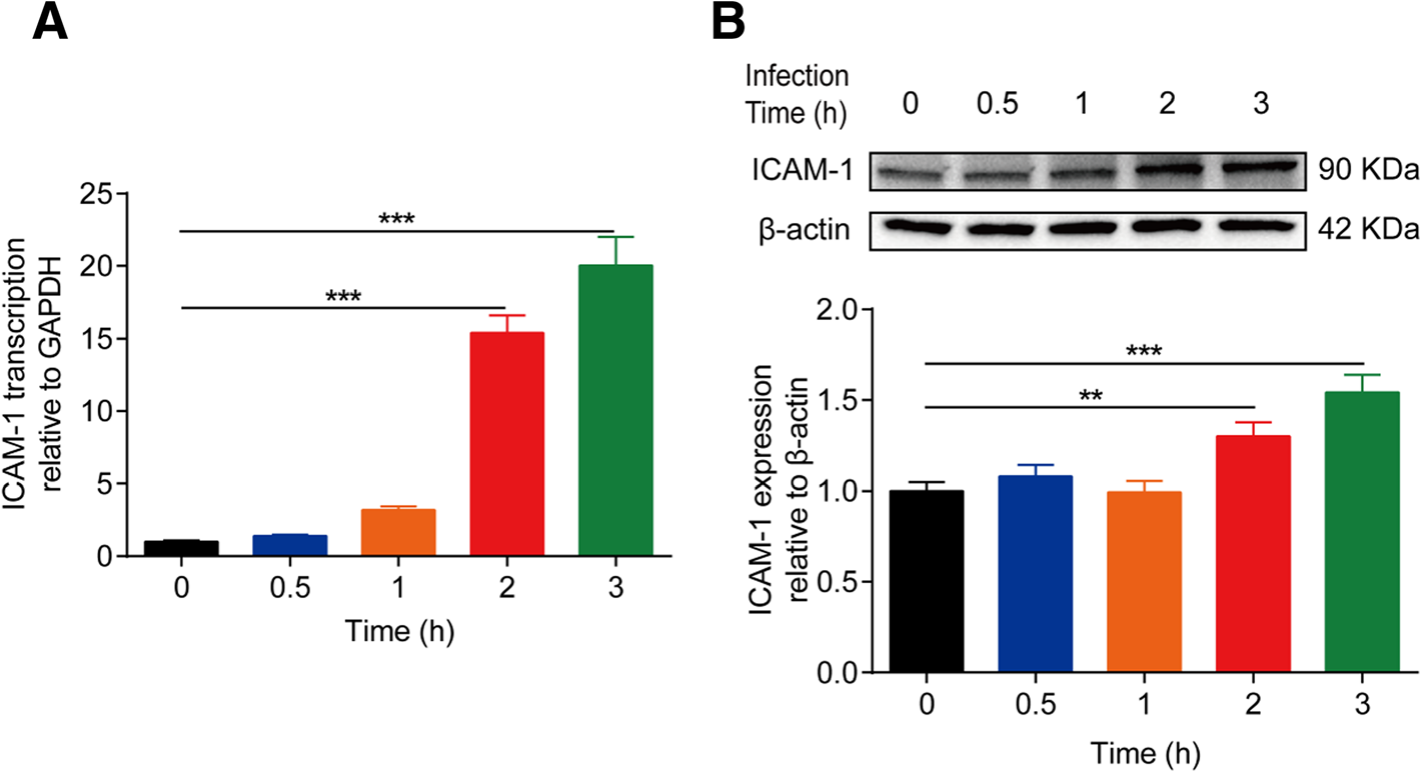
Meningitic *E. coli* induced significant upregulation of ICAM-1 in hBMECs. **a** PCN033 infection caused a significant upregulation of ICAM-1 in hBMECs time-dependently. GAPDH was used as the internal reference. **b** Western blot analysis revealed increased expression of ICAM-1 in hBMECs in response to infection. β-actin was used as the loading control, and densitometry was performed to analyze the differences

**Fig. 5:**
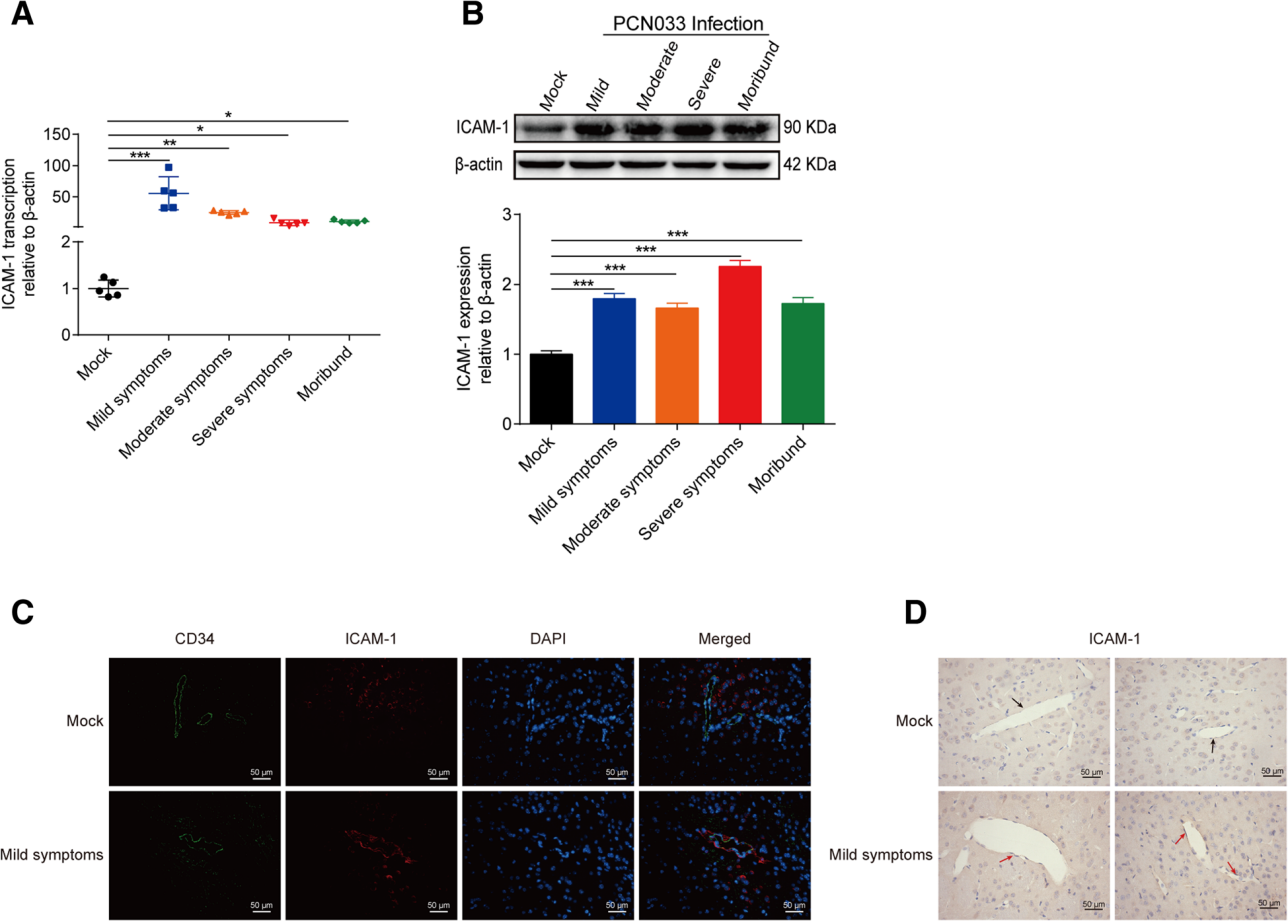
ICAM-1 expression was elevated in the brains of mice challenged by meningitic *E. coli*. **a** Real-time PCR determination of ICAM-1 transcription in brain total RNA from mice exhibiting different symptoms (*n* = 5). The transcription of β-actin was used as the internal reference. Data were presented as mean ± SD from five individual mice in each group. **b** Brain lysates from challenged mice with different signs were subjected to western blot analysis for expression of ICAM-1. β-actin expression was used as the loading control, and densitometry of the bands was performed to compare differences. **c** The brains of mild symptom and control mice were sectioned and analyzed for the expression of ICAM-1 via IF. The perivascular ICAM-1 was stained red. CD34 was specifically stained in green to label the blood vessel, and the cell nucleus was stained blue with DAPI. Scale bar indicates 50 μM. **d** The brains of mild symptom and control mice were subjected to ICAM-1 expression by IHC. ICAM-1 around the vessels was indicated by red arrows (infection group) or by black arrows (mock group). Scale bar indicates 50 μM

We also examined another important host molecule ICAM-1, which is widely recognized to be a biomarker for endothelial immunological activation [21]. Our previous transcriptomics data suggested that ICAM-1 was sharply increased in hBMECs upon meningitic *E. coli* infection [18]. In this work, we further verified this upregulation in vitro and in vivo. In hBMEC model, we found that mRNA transcription as well as the protein level of ICAM-1 showed a significant and time-dependent increase accompanying the infection (Fig. 4a, b). In challenged mice, the transcriptional level of ICAM-1 mRNA in mice brains was also significantly upregulated, with a greater than 50-fold increase at the early stage with mild symptoms (Fig. 5a). Meanwhile at the protein level, we also observed a significant increase of ICAM-1 in brains of the infected mice and maintained the higher expression in the course of infection (Fig. 5b). IF and IHC were additionally performed to ensure the overexpression of ICAM-1 in brains of the challenged mice with mild symptoms. As observed in Fig. 5c, ICAM-1 expression was found to be elevated and co-localized with blood vessels labeling with CD34 in the challenged mice, compared with the control group, and the same expression pattern was also observed by the IHC approach (Fig. 5d). These in vivo and in vitro evidences together indicate that meningitic *E. coli* challenge significantly induced ICAM-1 in endothelial cells.

### Blockage of ICAM-1 attenuates CNS inflammatory responses induced by meningitic *E. coli*

**Fig. 6:**
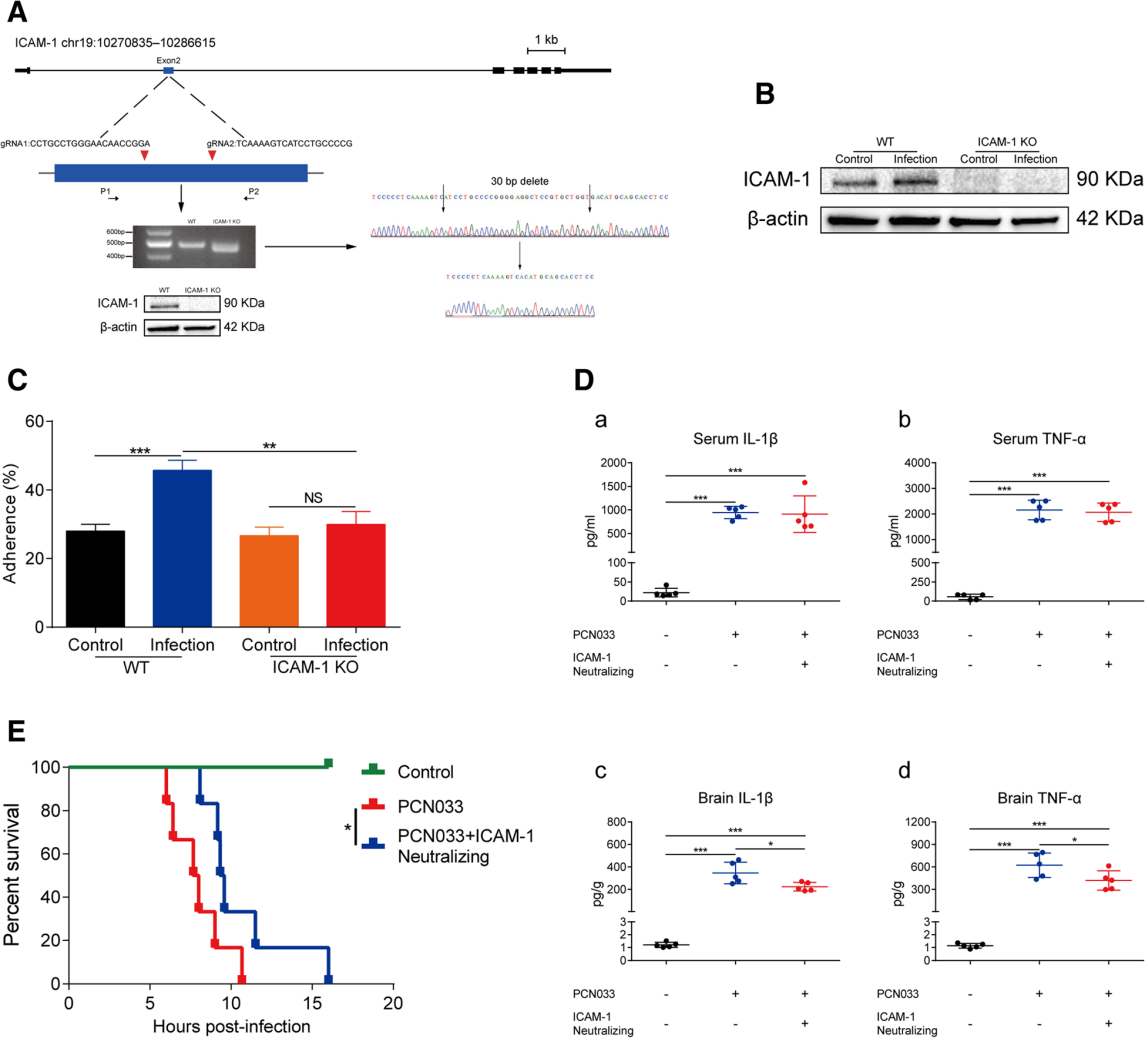
Blockage of ICAM-1 significantly attenuates meningitic *E. coli*-caused neuroinflammatory responses and extends the survival. **A** Schematics briefly showing the design of two gRNAs (gRNA1 and gRNA2) in the ICAM-1 Exon2 and the identification of the ICAM-1 knockout (KO) through PCR amplification, sequencing, and western blot analysis. Specific primers (P1 and P2) were used to analyze the ICAM-1 sequence in the genome. A total of 30 bp was deleted in ICAM-1 KO cells, in which the ICAM-1 protein was not expressed. **B** The induction of ICAM-1 by meningitic *E. coli* was completely abolished in hBMECs accompanying with the knockout of ICAM-1. **C** Meningitic *E. coli*-mediated increased monocytes (THP-1) adhesion to hBMECs was significantly blocked when ICAM-1 was knocked out. All data were presented as mean ± SD for three individual experiments. **D** MSD analysis of the IL-1β and TNF-α amount in serum and brain lysates from control mice, meningitic *E. coli*-challenged mice with or without ICAM-1-neutralizing antibody pretreatment. Data were expressed as mean ± SD (*n* = 5). **E** ICAM-1-neutralizing antibody pretreatment significantly prolongs mice survival time during bacterial challenge. Survival of mice in each group was monitored for 20 h after tail vein injection of meningitic *E. coli*. Data were collected and shown as Kaplan-Meier survival curves (*n* = 6)

Cell-to-cell interactions, such as adhesion of monocytes to endothelial cells or monocytes migration toward endothelial cells, are considered important events in initiating the inflammatory response [32]. We sought to investigate whether the increased expression of ICAM-1 in hBMECs is correlated with an enhanced association with monocytes, thus initiating the inflammatory response. Here, ICAM-1 was genetically destroyed in hBMECs using the clustered regularly interspaced short palindromic repeats (CRISPR)/Cas9 approach by introducing two small guide RNAs, and its deletion was validated by PCR amplification, western blot analysis, and sequencing (Fig. 6a). Meanwhile, the infection-induced upregulation of ICAM-1 in endothelial cells was completely abolished, without any ICAM-1 expression in the knockout cells (Fig. 6b). Moreover, we found significantly increased adherence of the THP-1 cells to the infected hBMECs, compared with that to the control cells, whereas this enhanced cell-cell adherence was completely blocked when ICAM-1 was knocked out (Fig. 6c), suggesting an ICAM-1-mediated neutrophils or monocyte recruitment in response to meningitic *E. coli* infection. In vivo, by using MSD Multi-Array® assay, we evaluated the contribution of ICAM-1 to the production of proinflammatory cytokines in response to the infection, by intravenous injection of the ICAM-1 neutralizing antibody prior to bacterial challenge. We observed that functional blockage of ICAM-1 by neutralizing antibody had no effects on the generation of IL-1β and TNF-α in serum, with the similar high levels in meningitic *E. coli* challenge mice with or without ICAM-1 neutralization (Fig. 6d). However, levels of both IL-1β and TNF-α in mice brains were significantly decreased when ICAM-1-neutralizing antibody was injected, even though there were still high levels of IL-1β and TNF-α in challenged mice brains with ICAM-1 neutralization, compared with that in the uninfected control mice (Fig. 6d), reflecting a certain contribution of ICAM-1 to the infection-induced neuroinflammatory response. Based on these observations, we next evaluated the potential protective effects of ICAM-1-neutralizing antibody to the lethal dosage of meningitic *E. coli* PCN033 challenge. All mice in the control groups (PBS) survived during the whole observation, and the PCN033-challenged mice died out gradually within around 10 h. Although all mice that had been injected with ICAM-1-neutralizing antibody eventually died, their survival time lasted longer than that of the mice without neutralizing antibody injection (Fig. 6e). Anyway, the current findings revealed an important role of ICAM-1 in directing the circulating monocytes or neutrophils migration toward the activated endothelium and partly facilitating the neuroinflammatory responses.

## Discussion

**Fig. 7:**
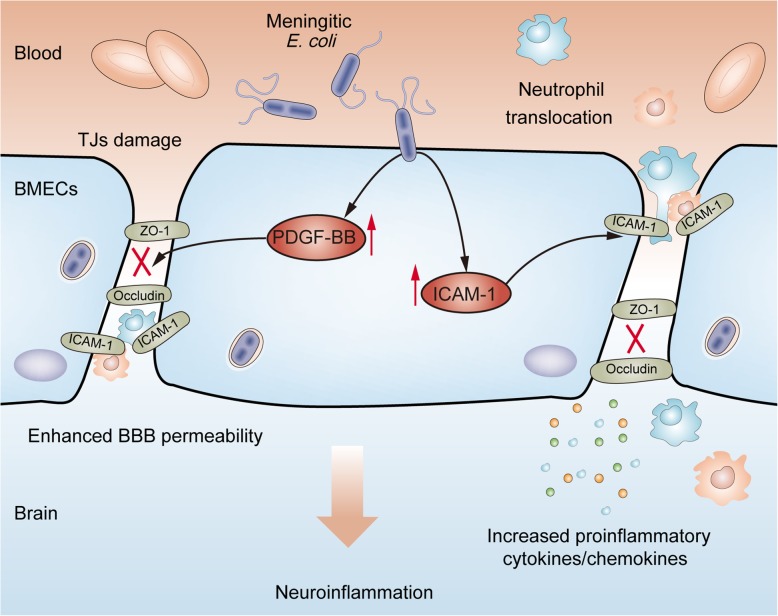
Schematic presentation of meningitic *E. coli* induction of PDGF-BB and ICAM-1 for BBB disruption as well as neuroinflammatory responses. Infection-induced PDGF-BB mediates the decrease of TJ proteins to enhance permeability, and infection-induced ICAM-1 expression in endothelial cells largely promotes circulating monocytes or neutrophils to traverse the BBB and magnify neuroinflammatory responses

Pathogenic *E. coli* is traditionally considered to be an exclusively intestinal-specific microorganism responsible for a range of digestive tract infections or diseases. However, an increasing number of studies have reported the infection as well as isolation of pathogenic *E. coli* strains from multiple extraintestinal tissues [33, 34], suggesting that these pathogenic *E. coli* strains are able to break through multiple host tissue barriers to cause infection [35, 36]. In our previous study, we reported the isolation and characterization of a meningitis-causing *E. coli* PCN033, which was isolated from the brain of diseased pig and was subsequently demonstrated to be able to invade the BBB and cause CNS dysfunction [25, 37]. Subsequently, we used RNA-sequencing to explore the potential host factors involved in this infection process [18], and herein, we laid emphasis on two host molecules, PDGF-BB and ICAM-1, which were significantly induced by the challenge of meningitic *E. coli* PCN033. With further in vivo and in vitro verification in response to infection, we demonstrated that meningitic *E. coli*-induced PDGF-BB negatively regulated the expression of TJ proteins including ZO-1, Occludin, and Claudin-5, enlarging endothelial permeability and causing BBB disruption. Meanwhile, the bacterial induction of ICAM-1 largely promoted the development of CNS inflammation and exacerbated the death (Fig. 7). These current findings largely support the notion that both PDGF-BB and ICAM-1 are two critical host targets that can be induced by meningitic *E. coli* for successful infection of the CNS.

As mentioned above, BMECs are tightly interconnected by the formation of TJ proteins, which restrict the crossing of pathogenic bacteria into the BBB. BBB dysfunction is widely considered a hallmark of the pathophysiology of bacterial meningitis [38], and several inflammatory-related mediators, such as vascular endothelial growth factor A (VEGFA), IL-1β, IL-6, IL-17A, TNF-α, nitric oxide, and matrix metalloproteinases (MMPs), have all been implicated in BBB disruption [39–42]. Numerous in vitro and in vivo studies have reported that meningitis-causing microbes can affect endothelial barrier integrity by degrading or redistributing TJ proteins. For example, recent studies have indicated that enolase, a potential virulence factor of *Streptococcus suis* serotype 2, can increase BBB permeability by promoting IL-8 secretion [43]. In *Neisseria*-induced meningitis, MMP-8 was involved in the proteolytic cleavage of the TJ protein Occludin [44]. Moreover, group B *Streptococcus* induction of Snail-1 was sufficient to facilitate TJ disruption, promoting BBB permeability to allow bacterial passage [2]. In *E. coli*-mediated meningitis, studies have demonstrated that meningitic *E. coli* can enhance BBB permeability by decreasing expression as well as redistributing TJ proteins [37, 45]. However, what are the involving host molecules that potently induced by meningitic *E. coli*, and their possible contributions during this process remain poorly understood.

PDGF-BB is known to play a critical role in the maintenance of CNS stability by controlling BBB homeostasis [46]. However, in addition to its neuroprotective effects, PDGF-BB has also been recognized as a vascular permeability factor that can lead to endothelial barrier dysfunction in an ischemic stroke model [47], Moreover, cocaine-mediated induction of PDGF-BB in hBMECs was reported to result in BBB damage by decreasing ZO-1 expression [48]. Here, our in vitro and in vivo data also demonstrated that meningitic *E. coli* induced the upregulation of PDGF-BB, which dose-dependently increased BBB permeability by decreasing the expression of TJ proteins. These findings therefore support the essential roles of cellular PDGF-BB in regulation of BBB permeability in meningitic *E. coli* challenge.

In addition to PDGF-BB, we also noted a significant induction of ICAM-1 in hBMECs in response to infection. The induction of endothelial ICAM-1 is widely accepted to be an important indicator of endothelial activation, and integrin LFA-1 has been reported to play a central role in T cell migration [49]. The general adhesion of endothelial ICAM-1 with LFA-1 in T cells initiates the circulating T cell transmigration through endothelial cells, mediating subsequent inflammatory responses [50], and previous study has also demonstrated that blocking of ICAM-1 via using a neutralizing antibody greatly decreased T cells migration, without any side effects on cell permeability [19]. In West Nile virus (WNV)-induced meningitis, inhibition of adhesion molecules in endothelial cells mainly contributed to the protection of the BBB, limiting neuroinflammation and virus-CNS entry [51]. In an early work on the interaction between *E. coli* and hBMECs, the outer membrane protein A of the *E. coli* K1 strain selectively mediated enhanced expression of ICAM-1 in hBMECs [23]. Here in the current study, we provided further evidences for the significant induction of ICAM-1 by meningitic *E. coli* strain in vivo and in vitro and elucidated the essential roles of ICAM-1 in mediating the migration of monocytes or neutrophils through the BBB, as well as the subsequent neuroinflammatory responses in the brain. Noticeably, we did observe a significant reduction of the proinflammatory cytokines IL-1β and TNF-α in the brains of mice receiving ICAM-1 neutralizing antibody; however, the expression level of these two cytokines in these mice were still greatly higher than those in control mice, suggesting that there must be some other host factors contributing to the neuroinflammation, except ICAM-1, and this might account for only the prolonged survival of the mice receiving neutralizing antibody, but not a complete protection. Taken together, although ICAM-1 is widely considered an indicator of endothelial activation, we believe it may also represent an important host target that can be induced by meningitic *E. coli* during infection.

An important factor in the development of meningitis is the level of bacteremia [52]. After hematogenous infection, pathogenic bacteria propagate in the bloodstream and effectively avoid complement or macrophage-mediated killing and clearance in the blood, only in such a way the circulating bacteria get opportunity to enter the brain, invade the BBB, and induce CNS disorder [53]. Obviously, it is not the case that pathogenic bacteria will definitely cross the BBB to cause CNS infection when entering the blood, but on the contrary, if pathogenic bacteria invade brain tissues and cause certain phenotypic symptoms correspondingly, it must mean that the pathogenic bacteria can survive in the blood, escape the clearance, and then invade the BBB. In our study, we observed the acute death of the mice within 12 h of infection, which is likely resulted from an increasing bacteremia and subsequently died from severe sepsis. However, it cannot be ruled out that there are indeed functional changes of the BBB and CNS inflammatory disorders during this process, which further aggravates the death process of the challenged mice. Therefore, on the one hand, we have observed the acute death of mice post infection, and on the other hand, we have also demonstrated the BBB disruption as well as neuroinflammatory disorder of dead or dying animals in this acute period, in which both PDGF-BB and ICAM-1 do play important roles.

## Conclusions

In this study, the current findings highlight the importance of host cellular PDGF-BB and ICAM-1 in meningitic *E. coli*-induced BBB disruption, as well as CNS inflammatory responses (Fig. 7), which are the two essential components determining the occurrence and development of CNS infection and damage. Meningitic *E. coli* infection could enhance BBB permeability via PDGF-BB-mediated TJ breakdown, and the infection-induced endothelial activation by ICAM-1 upregulation largely initiates the development of CNS inflammatory responses. Our findings strongly support the functional involvement of PDGF-BB and ICAM-1 in meningitic *E. coli* invasion of the BBB, which represent important targets for further prevention and therapy of CNS disorders resulting from meningitis-causing pathogens.

## Abbreviations

96W1E+: 96-well chamber slides
BBB: Blood-brain barrier
CFU: Colony-forming units
CNS: Central nervous system
CRISPR: Clustered regularly interspaced short palindromic repeats
DAB: Diaminobenzidine
*E. coli*: *Escherichia coli*
ECIS: Electric cell-substrate impedance sensing
ECL: Electrochemiluminescence
ELISA: Enzyme-linked immunosorbent assay
FBS: Fetal bovine serum
hBMECs: Human brain microvascular endothelial cells
ICAM-1: Intercellular adhesion molecule-1
IF: Immunofluorescence
IHC: Immunohistochemistry
IL-1β: Interleukin-1 beta
IL-6: Interleukin-6
IL-8: Interleukin-8
MCP-1: Monocyte chemoattractant protein-1
MMPs: Matrix metalloproteinases
PBS: Phosphate-buffered saline
PDGF-B: Platelet-derived growth factor-B
SPF: Specific pathogen free
TEER: Trans-endothelial electric resistance
TJ: Tight junction
TNFα: Tumor necrosis factor-alpha
VEGFA: Vascular endothelial growth factor A
WNV: West Nile virus
ZO-1: Zonala occludin-1

## References

[1] Kim KS (2008). Mechanisms of microbial traversal of the blood-brain barrier. Nat Rev Microbiol.

[2] Kim BJ, Hancock BM, Bermudez A, Del Cid N, Reyes E, van Sorge NM, Lauth X, Smurthwaite CA, Hilton BJ, Stotland A (2015). Bacterial induction of Snail1 contributes to blood-brain barrier disruption. J Clin Invest.

[3] Wang X, Maruvada R, Morris AJ, Liu JO, Wolfgang MJ, Baek DJ, Bittman R, Kim KS (2016). Sphingosine 1-phosphate activation of EGFR as a novel target for meningitic *Escherichia coli* penetration of the blood-brain barrier. Plos Pathog.

[4] Van Sorge NM, Doran KS (2012). Defense at the border: the blood-brain barrier versus bacterial foreigners. Future Microbiol.

[5] Liu WT, Lv YJ, Yang RC, Fu JY, Liu L, Wang H, Cao Q, Tan C, Chen HC, Wang XR. New insights into meningitic *Escherichia coli* infection of brain microvascular endothelial cells from quantitative proteomics analysis. J Neuroinflammation. 2018;15:291. 10.1186/s12974-018-1325-z PMC6195690 30340642

[6] Kim KS (2003). Pathogenesis of bacterial meningitis: From bacteraemia to neuronal injury. Nat Rev Neurosci.

[7] Burkhart A, Thomsen LB, Thomsen MS, Lichota J, Fazakas C, Krizbai I, Moos T (2015). Transfection of brain capillary endothelial cells in primary culture with defined blood-brain barrier properties. Fluids Barriers CNS.

[8] Dejana E, Tournier-Lasserve E, Weinstein BM (2009). The control of vascular integrity by endothelial cell junctions: molecular basis and pathological implications. Dev Cell.

[9] Candelario-Jalil E, Yang Y, Rosenberg GA (2009). Diverse roles of matrix metalloproteinases and tissue inhibitors of metalloproteinases in neuroinflammation and cerebral ischemia. Neuroscience.

[10] Andrae J, Gallini R, Betsholtz C (2008). Role of platelet-derived growth factors in physiology and medicine. Gene Dev.

[11] Funa K, Sasahara M (2014). The roles of PDGF in development and during neurogenesis in the normal and diseased nervous system. J Neuroimmune Pharm.

[12] Wen HX, Lu YM, Yao HH, Buch S. Morphine induces expression of platelet-derived growth factor in human brain microvascular endothelial cells: implication for vascular permeability. Plos One. 2011;6:e21707. 10.1371/journal.pone.0021707 PMC3125302 21738771

[13] Keller A, Westenberger A, Sobrido MJ, Garcia-Murias M, Domingo A, Sears RL, Lemos RR, Ordonez-Ugalde A, Nicolas G, da Cunha JE (2013). Mutations in the gene encoding PDGF-B cause brain calcifications in humans and mice. Nat Genet.

[14] Mittapalli RK, Chung AH, Parrish KE, Crabtree D, Halvorson KG, Hu G, Elmquist WF, Becher OJ (2016). ABCG2 and ABCB1 limit the efficacy of dasatinib in a PDGF-B-driven brainstem glioma model. Mol Cancer Ther.

[15] Wasmuth HE, Tag CG, Van de Leur E, Hellerbrand C, Mueller T, Berg T, Puhl G, Neuhaus P, Samuel D, Trautwein C (2009). The marburg I variant (G534E) of the factor VII-activating protease determines liver fibrosis in hepatitis C infection by reduced proteolysis of platelet-derived growth factor BB. Hepatology.

[16] Yao H, Bethel-Brown C, Niu F, Yang L, Peng F, Buch S (2014). Yin and Yang of PDGF-mediated signaling pathway in the context of HIV infection and drug abuse. J Neuroimmune Pharmacol.

[17] Miners JS, Schulz I, Love S. Differing associations between Abeta accumulation, hypoperfusion, blood-brain barrier dysfunction and loss of PDGFRB pericyte marker in the precuneus and parietal white matter in Alzheimer's disease. J Cereb Blood Flow Metab. 2018;38:103–15. 10.1177/0271678X17690761 PMC5757436 28151041

[18] Yang R, Huang F, Fu J, Dou B, Xu B, Miao L, Liu W, Yang X, Tan C, Chen H, Wang X (2016). Differential transcription profiles of long non-coding RNAs in primary human brain microvascular endothelial cells in response to meningitic *Escherichia coli*. Sci Rep.

[19] Wong D, Prameya R, Dorovini-Zis K (2007). Adhesion and migration of polymorphonuclear leukocytes across human brain microvessel endothelial cells are differentially regulated by endothelial cell adhesion molecules and modulate monolayer permeability. J Neuroimmunol.

[20] Lee W, Ku SK, Bae JS (2014). Vascular barrier protective effects of orientin and isoorientin in LPS-induced inflammation in vitro and in vivo. Vascul Pharmacol.

[21] Lopes Pinheiro MA, Kroon J, Hoogenboezem M, Geerts D, van Het Hof B, van der Pol SM, van Buul JD, de Vries HE (2016). Acid sphingomyelinase-derived ceramide regulates ICAM-1 function during T cell transmigration across brain endothelial cells. J Immunol.

[22] Tripathi AK, Sullivan DJ, Stins MF (2006). Plasmodium falciparum-infected erythrocytes increase intercellular adhesion molecule 1 expression on brain endothelium through NF-kappaB. Infect Immun.

[23] Selvaraj SK, Periandythevar P, Prasadarao NV (2007). Outer membrane protein A of *Escherichia coli* K1 selectively enhances the expression of intercellular adhesion molecule-1 in brain microvascular endothelial cells. Microbes Infect.

[24] Conzemius R, Ganjian H, Blaas D, Fuchs R (2016). ICAM-1 binding *rhinoviruses* A89 and B14 uncoat in different endosomal compartments. J Virol.

[25] Liu CY, Zheng HJ, Yang MJ, Xu ZF, Wang XR, Wei LY, Tang B, Liu F, Zhang YY, Ding Y, et al. Genome analysis and in vivo virulence of porcine extraintestinal pathogenic *Escherichia coli* strain PCN033. BMC Genomics. 2015;16:717. 10.1186/s12864-015-1890-9 PMC4578781 26391348

[26] Stins MF, Badger J, Sik Kim K (2001). Bacterial invasion and transcytosis in transfected human brain microvascular endothelial cells. Microb Pathog.

[27] Li P, Wang R, Dong W, Hu L, Zong B, Zhang Y, Wang X, Guo A, Zhang A, Xiang Y (2017). Comparative proteomics analysis of human macrophages infected with virulent *Mycobacterium bovis*. Front Cell Infect Microbiol.

[28] Yang RC, Xu BJ, Yang B, Fu JY, Liu L, Amjad N, Cai AL, Tan C, Chen HC, Wang XR (2018). Circular RNA transcriptomic analysis of primary human brain microvascular endothelial cells infected with meningitic *Escherichia coli*. Mol Ther Nucleic Acids.

[29] Zhu L, Maruvada R, Sapirstein A, Malik KU, Peters-Golden M, Kim KS (2010). Arachidonic acid metabolism regulates *Escherichia coli* penetration of the blood-brain barrier. Infect Immun.

[30] Li F, Wang Y, Yu L, Cao S, Wang K, Yuan J, Wang C, Wang K, Cui M, Fu ZF (2015). Viral infection of the central nervous system and neuroinflammation precede blood-brain barrier disruption during *Japanese encephalitis* virus infection. J Virol.

[31] Bae JW, Bae JS (2011). Barrier protective effects of lycopene in human endothelial cells. Inflamm Res.

[32] Hansson GK, Libby P (2006). The immune response in atherosclerosis: a double-edged sword. Nat Rev Immunol.

[33] Chakraborty A, Saralaya V, Adhikari P, Shenoy S, Baliga S, Hegde A (2015). Characterization of *Escherichia coli* phylogenetic groups associated with extraintestinal infections in South Indian population. Ann Med Health Sci Res.

[34] Russo TA, Johnson JR (2000). Proposal for a new inclusive designation for extraintestinal pathogenic isolates of *Escherichia coli*: ExPEC. J Infect Dis.

[35] Zeng Q, He X, Puthiyakunnon S, Xiao H, Gong Z, Boddu S, Chen L, Tian H, Huang SH, Cao H (2017). Probiotic mixture golden bifido prevents neonatal *Escherichia coli* K1 translocation via enhancing intestinal defense. Front Microbiol.

[36] Poole NM, Green SI, Rajan A, Vela LE, Zeng XL, Estes MK, Maresso AW. Role for FimH in extraintestinal pathogenic *Escherichia coli* invasion and translocation through the intestinal epithelium. Infect Immun. 2017;85:e00581–17. 10.1128/IAI.00581-17 PMC5649017 28808163

[37] Yang R, Liu W, Miao L, Yang X, Fu J, Dou B, Cai A, Zong X, Tan C, Chen H, Wang X (2016). Induction of VEGFA and Snail-1 by meningitic *Escherichia coli* mediates disruption of the blood-brain barrier. Oncotarget.

[38] Winger RC, Koblinski JE, Kanda T, Ransohoff RM, Muller WA (2014). Rapid remodeling of tight junctions during paracellular diapedesis in a human model of the blood-brain barrier. J Immunol.

[39] McMillin MA, Frampton GA, Seiwell AP, Patel NS, Jacobs AN, DeMorrow S (2015). TGFbeta1 exacerbates blood-brain barrier permeability in a mouse model of hepatic encephalopathy via upregulation of MMP9 and downregulation of claudin-5. Lab Invest.

[40] Miao Z, Dong Y, Fang W, Shang D, Liu D, Zhang K, Li B, Chen YH (2014). VEGF increases paracellular permeability in brain endothelial cells via upregulation of EphA2. Anat Rec.

[41] Rochfort KD, Collins LE, McLoughlin A, Cummins PM (2016). Tumour necrosis factor-alpha-mediated disruption of cerebrovascular endothelial barrier integrity in vitro involves the production of proinflammatory interleukin-6. J Neurochem.

[42] Ni PF, Dong HQ, Wang YW, Zhou Q, Xu MM, Qian YN, Sun J. IL-17A contributes to perioperative neurocognitive disorders through blood-brain barrier disruption in aged mice. J Neuroinflammation. 2018;15:332. 10.1186/s12974-018-1374-3 PMC6267879 30501622

[43] Sun Y, Li N, Zhang J, Liu H, Liu J, Xia X, Sun C, Feng X, Gu J, Du C (2016). Enolase of *Streptococcus suis* serotype 2 enhances blood-brain barrier permeability by inducing IL-8 release. Inflammation.

[44] Schubert-Unkmeir A, Konrad C, Slanina H, Czapek F, Hebling S, Frosch M (2010). *Neisseria meningitidis* induces brain microvascular endothelial cell detachment from the matrix and cleavage of occludin: a role for MMP-8. Plos Pathog.

[45] Sukumaran SK, Prasadarao NV (2003). *Escherichia coli* K1 invasion increases human brain microvascular endothelial cell monolayer permeability by disassembling vascular-endothelial cadherins at tight junctions. J Infect Dis.

[46] Kastin AJ, Akerstrom V, Hackler L, Pan WH. Different mechanisms influencing permeation of PDGF-AA and PDGF-BB across the blood-brain barrier. J Neurochem. 2003;87:7–12. 10.1046/j.1471-4159.2003.01933.x 12969247

[47] Su EJ, Fredriksson L, Geyer M, Folestad E, Cale J, Andrae J, Gao Y, Pietras K, Mann K, Yepes M (2008). Activation of PDGF-CC by tissue plasminogen activator impairs blood-brain barrier integrity during ischemic stroke. Nat Med.

[48] Yao H, Duan M, Buch S (2011). Cocaine-mediated induction of platelet-derived growth factor: implication for increased vascular permeability. Blood.

[49] Lee YW, Lee WH (2008). Protective effects of genistein on proinflammatory pathways in human brain microvascular endothelial cells. J Nutr Biochem.

[50] Verma NK, Fazil MH, Ong ST, Chalasani ML, Low JH, Kottaiswamy A, Praseetha P, Kizhakeyil A, Kumar S, Panda AK (2016). LFA-1/ICAM-1 ligation in human T cells promotes Th1 polarization through a GSK3β signaling-dependent notch pathway. J Immunol.

[51] Roe K, Orillo B, Verma S (2014). West Nile virus-induced cell adhesion molecules on human brain microvascular endothelial cells regulate leukocyte adhesion and modulate permeability of the in vitro blood-brain barrier model. Plos One.

[52] Coureuil M, Lecuyer H, Bourdoulous S, Nassif X (2017). A journey into the brain: insight into how bacterial pathogens cross blood-brain barriers. Nat Rev Microbiol.

[53] Kim KS. Human meningitis-associated *Escherichia coli*. EcoSal Plus. 2016;7. 10.1128/ecosalplus. 10.1128/ecosalplus.esp-0015-2015 PMC4881430 27223820

